# The ecoepidemiology of cutaneous leishmaniasis in Ethiopia: a systematic review and meta-analysis

**DOI:** 10.1186/s13071-026-07376-3

**Published:** 2026-05-09

**Authors:** Galana Mamo Ayana, Derese Bekele Daba, Bedilu Alamirie Ejigu, Debisa Eshatu Wendimu, Merga Belina, Eshetu Molla, Elifaged Hailemeskel, Adisu Asefa, Girma Shumie, Tedros Nigusse, Mourad Barhoumi, Souheila Guerbouj, Salma Feki Ben-Salah, Temesgen Tafesse, Henock Bekele Keto, Mulugeta Woldemeskel, Teklu Cherkose, Sagni Chali Jira, Jihenne Ben Aissa-Haj, Elisa Sicuri, Ikram Guizani, Endalamaw Gadisa

**Affiliations:** 1 Malaria and Neglected Tropical Diseases, Armauer Hansen Research Institute, Addis Ababa, Ethiopia; 2 Department of Statistics, Addis Ababa University, Addis Ababa, Ethiopia; 3 Department of Statistics, CDT-Africa, Addis Ababa University, Addis Ababa, Ethiopia; 4 Laboratory of Molecular Epidemiology and Experimental Pathology-LR16IPT04, Institut Pasteur de Tunis, University of Tunis El Manar, Tunis, Tunisia; 5 Laboratory of Viruses, Vectors and Hosts-LR20IPT02, Institut Pasteur de Tunis, University of Tunis El Manar, Tunis, Tunisia; 6 Neglected Tropical Diseases Case Management, World Health Organization, Addis Ababa, Ethiopia; 7 Department of Human and Experimental Pathology, Institut Pasteur de Tunis, 1002 Tunis, Tunisia; 8 Laboratory of Biomedical Genomics and Oncogenetics, Institut Pasteur de Tunis, University of Tunis El Manar, 1002 Tunis, Tunisia; 9 Instituto de Salud Global de Barcelona, Barcelona, Spain

**Keywords:** Cutaneous leishmaniasis, Pooled prevalence, Risk factors, Ecoepidemiology, Access to care, Ethiopia

## Abstract

**Background:**

Cutaneous leishmaniasis (CL) is a neglected tropical skin disease. In Ethiopia, CL is a public health concern; about 29 million people are at risk, with an estimated incidence of up to 50,000 new cases annually. In endemic communities, access to diagnosis, treatment, and prevention is crucial but often limited. Understanding the prevalence and access to care, as well as exploring its relationship to agroecological factors, is crucial to inform control strategies.

**Objectives:**

The aim of this work is to estimate the pooled prevalence, access to care service facilities, and spatial distribution and relationship to agroecological factors.

**Methods:**

A systematic review and meta-analysis were conducted following the Preferred Reporting Items for Systematic Review and Meta-Analysis (PRISMA) framework. The metafor and metadata packages from R Studio were used to obtain pooled prevalence and odds ratio using a random-effects model with a double arcsine transformation. The CL endemicity at the woreda level was overlaid with the locations of CL treatment centers and agroecological zones using ArcGIS.

**Results:**

The pooled prevalence of CL was 6.75% (95% confidence interval (CI) 3.37–11.17). Sociodemographic factors (male gender, rural living) and environmental factors (muddy walls, outdoor sleeping, proximity to rocky habitats, and hyrax populations) were significantly associated with CL. CL cases were reported from 85 woredas with a broad spatial distribution; a higher proportion of them were from the Weyna Dega, Dega, and Upper Kola agroecological zones. Access to care is generally poor, with service centers for CL often centralized at the zone level.

**Conclusions:**

The estimated pooled prevalence likely underrepresents the true burden of CL. The identified risk factors are more related to rural livelihoods and living conditions, and most of the endemic woredas are in the most productive agrarian agroecological zones, which underlines the health and socioeconomic significance of CL in Ethiopia. Thus, decentralizing healthcare services and improving surveillance for CL are crucial steps in breaking the vicious cycle of poverty.

**Graphical Abstract:**

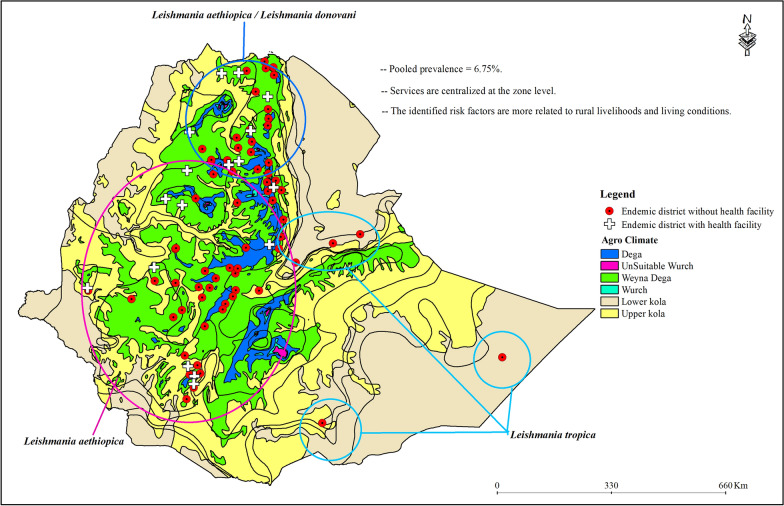

**Supplementary Information:**

The online version contains supplementary material available at 10.1186/s13071-026-07376-3.

## Background

Cutaneous leishmaniasis (CL) is a skin-related neglected tropical disease (NTD) endemic in over 70 countries [1, 2]. It is a major public health concern in the Eastern Mediterranean and African regions of the World Health Organization (WHO) [2–4]. It is often underreported; of the estimated global incidence of 0.7–1.2 million, only about 200,000 are reported to the WHO [1, 5]. Moreover, CL carries a significant psychosocial and economic burden, exacerbating existing vulnerability in marginalized communities [6].

Ethiopia is among the ten countries with high CL burden [7]. It is estimated that over 29 million live at risk, with an estimated incidence of up to 50,000 new cases annually, yet only a fraction (less than 800) are reported to the WHO [5]. Surveillance hardly exists, though CL has been described since 1913 and is well-known by several vernacular names, including Shahign, Chewie, Kunchir, Finchoftu, Gizwa, and Volbo in different endemic communities [8–10]. The most described foci, since the 1960s, are those in the highlands at 1600–2700 m above sea level (masl) [11, 12], while there are recent reports from the lowlands showing that the true distribution of the disease is far more extensive than presently recognized [13]. Outbreaks in areas hitherto not known to be endemic are not rare, including between 2003 and 2005 in Silti District, Silte Zone, Central Ethiopia regional state [14] and by 2020, in the Ankesha-Guagsa District, Awi Zone of the Amhara Regional State [15] and by 2023 in the Somali Regional State.

In 1973, Bray identified the causative parasite *Leishmania* *aethiopica*, the vectors, *Phlebotomus* *longipes* and *Ph. pedifer*, and reservoirs, *Procavia habessinica* and *Heterohyrax brucei* [16]. In 2023, a CL case attributed to *L. donovani* was reported in northern Ethiopia [17]. Furthermore, the CL case report in 2006 from Awash [18] and the outbreak in the Somali region in 2023 were associated with *L. tropica* [19]. The potential nonhuman reservoirs for *L. tropica* are bats (*Rhinolophus fumigatus* and *Triaenops afer*) and rodents (*Arvicanthis dementias* and *Mastomys awashensis*) [20, 21].

The Weyna Dega (the warm temperate highland) and Dega (cool temperate highland) areas, characterized by rocky cliffs, caves, crevices, and bushes, are favorable habitats for both sand flies and hyraxes, an area dominated by *L. aethiopica* [22, 23]. Arid areas, characterized by rocky crevices and animal burrows, are the habitats for rodents, bats, and sand flies, where *L. tropica* thrives [24–26].

To the best of our knowledge, there is no reliable and current estimate on the prevalence and risk factor distribution of CL in the different agroecological zones of Ethiopia. By bridging this knowledge gap, the ecoepidemiology and interplay between CL cases, vectors, and treatment centers distribution will facilitate the design of tailored control. Therefore, this review aimed to determine the pooled prevalence, identify the risk factors distribution, and indicate the spatial distribution of CL, vectors, and reservoir hosts with respect to the agroecology and health facilities treating CL cases in Ethiopia.

## Methods

### Protocol and registration

**Fig. 1:**
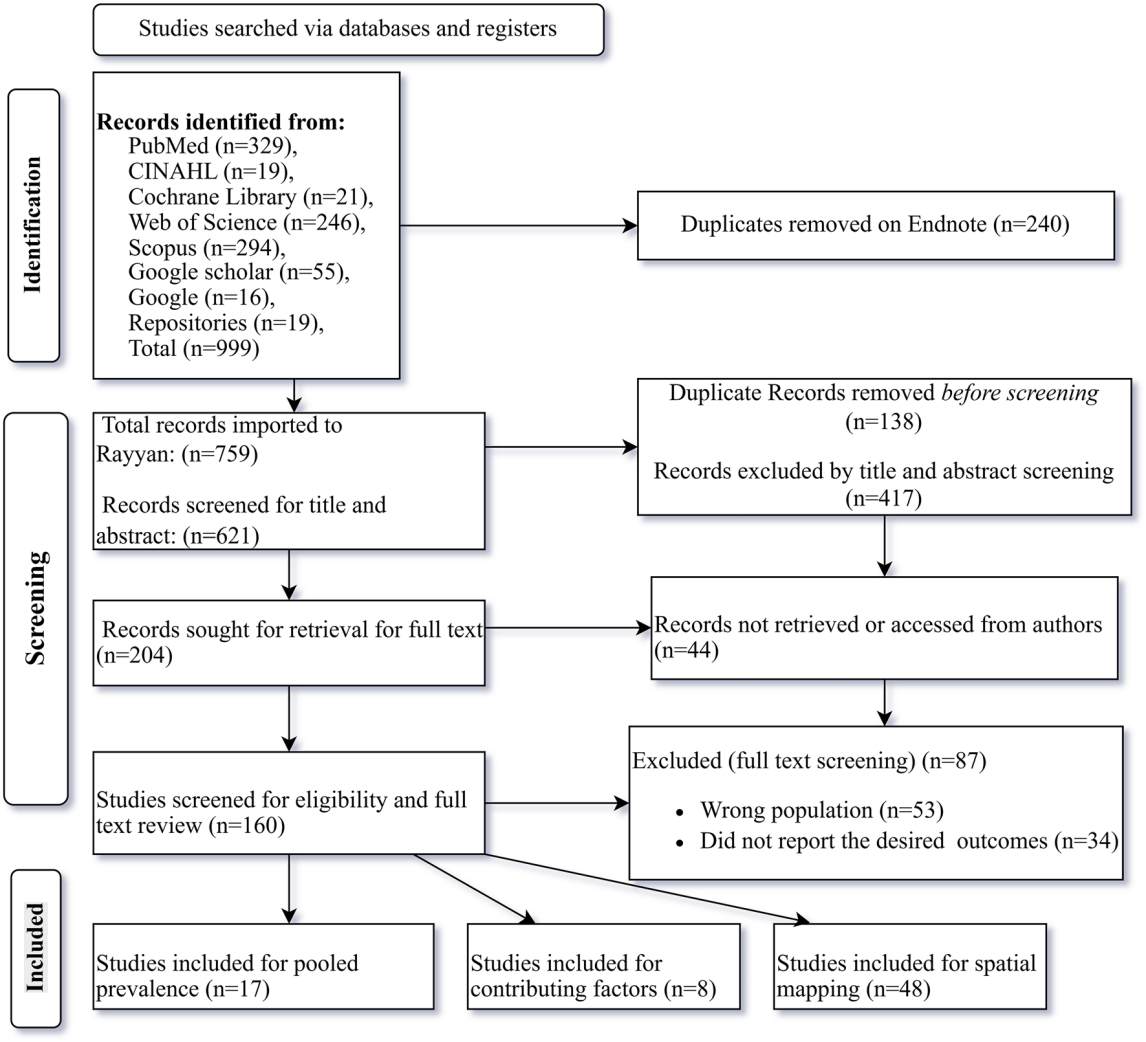
PRISMA flow diagram for new systematic reviews, which included searches of databases and registers

The protocol was registered in the PROSPERO database with no. CRD42024571850 [27] and is reported using the Preferred Reporting Items for Systematic Review and Meta-Analysis (PRISMA) checklist 2020 [28] (Fig. 1).

### Study area, period, and design

A systematic review and meta-analysis (SRMA) of published and unpublished documents on CL was conducted from its first notification (1913) in Ethiopia till October 2024. The Federal Democratic Republic of Ethiopia has 12 regional states and 2 city administrations. The country has four recognized administrative units: regional states, zones, woredas (also called districts), and Kebeles, the lowest administrative unit. The population size of a woreda varies; rural woredas typically have 80,000–120,000 people [29].

Ethiopia’s six agroecological zones vary in altitude and climate, strongly influencing agricultural productivity. The Kur zone (above 3700 masl) is cold, dry, and windy, limiting farming mostly to hardy crops such as barley and potatoes, with livestock such as sheep and goats. The Wurch zone (3200–3700 masl) is cold and humid with high rainfall, supporting moderate production of barley and wheat despite frost risks. The Dega zone (2400–3200 masl) has a cool, humid climate and rainfall between 900 and 1200 mm, favoring mixed farming of cereals. The Weyna Dega zone (1500–2300 masl) has moderate temperatures and bimodal rainfall, making it one of the most productive zones with diverse crops such as teff, maize, sorghum, pulses, and oilseeds. The Upper Kola zone (500–1500 masl) is semi-arid with variable rainfall, where drought-tolerant crops and livestock dominate. Lastly, the Lower Kola zone (below 500 masl), also called Bereha, is hot and dry with highly variable rainfall, resulting in low productivity focused on drought-resistant crops and pastoralism [30–33].

Ethiopia has a three-tier healthcare system. The primary tier includes health posts, health centers, and primary hospitals, serving about 5000, 25,000, and 100,000 people, respectively. The secondary tier has general hospitals serving up to 1.5 million people, and the tertiary tier has specialized hospitals serving up to 5 million. The woredas, in rural areas, are typical primary healthcare hubs aimed at improving accessible healthcare [34].

### Inclusion criteria

We included primary (original) studies (published or unpublished articles, theses, or dissertations) or reports from government or WHO, on CL cases, sand fly vectors, reservoir hosts, and associated risk factors, methodological surveys (cross sectional), case reports or series, short communications, and observational studies reporting associated factors that were written in English or any of the languages spoken in Ethiopia and conducted in geographically recognized Ethiopian areas.

### Exclusion criteria

We excluded studies or reports that did not indicate the target area (administrative boundaries at least to district level and/or outcome variables of CL prevalence and associated factors), or with incomplete data; cross-sectional studies focusing on Knowldge, Attitude and Practice (KAP); experimental studies that did not explicitly explain the geographical location and disease ascertainment; books or book chapters intending to provide scientific knowledge, review articles, or editorial letters without original data; studies with methodological limitations regarding either the outcome or exposure variables; and abstract-only articles after attempts to reach the corresponding author.

### Information sources and search strategy

A comprehensive literature search was conducted using PubMed, Scopus, CINAHL, and the Cochrane Library following each database’s specific search strategy. Additionally, a manual search was performed using Google and Google Scholar with a predefined set of search terms. In addition, unpublished primary data sources (gray literature) such as reports, dissertations, conference proceedings, government documents, and other reports were searched manually from the organizations’ websites and/or through contact with the responsible parties.

All identified articles were uploaded to EndNote version 20 to collate and remove duplicates. Citation details of potentially eligible studies were retrieved in full and imported into Rayyan. Two independent reviewers (D.B. and D.E.) screened the titles and abstracts; the full text of selected articles was critically appraised by independent reviewers (E.H. and E.M.). Reasons for exclusion and inclusion were recorded and reported. A third team of reviewers (G.M.A. and A.A.) was consulted to resolve disagreements at each stage of the screening. The screening process and results of the research are fully reported in the final review work and presented in the PRISMA flow diagram.

Patient, population, or problem: Studies involving patients (adults or children) with CL (with any type, such as localized, diffused, or mucocutaneous) who are from Ethiopia or with history of travel to Ethiopia were the primary focus. In addition, the search included all publications related to CL, reservoir hosts, sand flies, and CL–sand flies–reservoir hosts.

Intervention or exposure: Primary studies reporting environmental, social, or host-related CL exposure status in Ethiopia.

Outcome: The magnitude/prevalence/proportion/incidence of CL and risk factors associated with CL were measured by the odds ratio (OR). The presence of CL in the woreda, agroecological characteristics, service centers treating CL, and reservoir–vector distributions are presented using maps.

### Data extraction and quality assessment procedures

For quality checking, after screening, each selected article was independently reviewed for quality and risk of bias by two independent reviewers. Quality and risk of bias in the design of quantitative studies were assessed using the Joanna Briggs Institute (JBI) critical appraisal checklist.

For data extraction, two reviewers extracted data independently using a predesigned data extraction Excel form for each of the themes. The reviewers thoroughly examined the retrieved data for consistency, cleanness, and reliability. Disagreements in data extraction were settled among the two reviewers in the presence of a third reviewer.

### Data processing analysis

The extracted data were exported to RStudio version 4.4.2 for statistical analysis. The Der Simonian and Laird random effects model with double arcsine transformation was used for pooled prevalence with a 95% confidence interval. The Cochran *Q* test (*χ*^2^) and *I*^2^ statistics were used to detect and quantify heterogeneity, respectively, during the meta-analysis. The Cochran *Q* test (*P* ≤ 0.1) and *I*^2^ = 50% were the cutoff points used to detect heterogeneity and conduct further moderator analysis. To reduce the influence of random variations between the included studies, the potential source of heterogeneity was assessed using subgroup analysis. Visual inspection of funnel plots and Egger’s regression test were used to assess for publication bias.

To assess the influence of outlier studies on the pooled proportion, we screened for externally Studentized residuals (Additional file 1: Table S1). Studies with a *z*-value greater than ±2 were considered to have a potential impact on the overall result.

As the summary effect measure, the pooled odds ratio (OR) with 95% confidence interval (CI) was employed. Statistical significance was established at*P* < 0.05. *I*^2^ statistics were used to evaluate the heterogeneity of the studies. A random-effects model was employed where there was significant heterogeneity (*I*^2^ ≥ 50%), whereas a fixed-effect model was employed when there was little heterogeneity (*I*^2^ < 50%). The presence of CL was mapped at the woreda level. The endemic woreda map was then overlaid with the country’s agroecological zones. The list of CL treatment centers obtained from the WHO Ethiopian Country Office was overlaid with the endemic woredas. A map showing the distribution of CL vectors and reservoir species was also overlaid with agroecological zones. Shapefiles for administrative boundaries were obtained from the Ethiopian Central Statistics Service (CSA), and ecological zones were obtained from the Ethiopian Central Statistics Service and the Ministry of Agriculture geospatial database. Data on treatment center locations were retrieved from the WHO data. Mapping and spatial analysis were performed using ArcGIS 10.7.1

## Results

### Search results

A total of 73 articles were selected for full-text extraction; among these, 48, 17, and 8 articles contributed to woreda-level mapping, pooled prevalence, and the identification of contributing factors for CL in Ethiopia, respectively. Additionally, 37 more woredas were included from the field visit data mapping study, meaning that 85 woredas were considered in the mapping of endemic woredas. For mapping the CL treatment center, the WHO report on the treatment of CL from 2017 to 2024 was used.

### Prevalence of CL

**Fig. 2:**
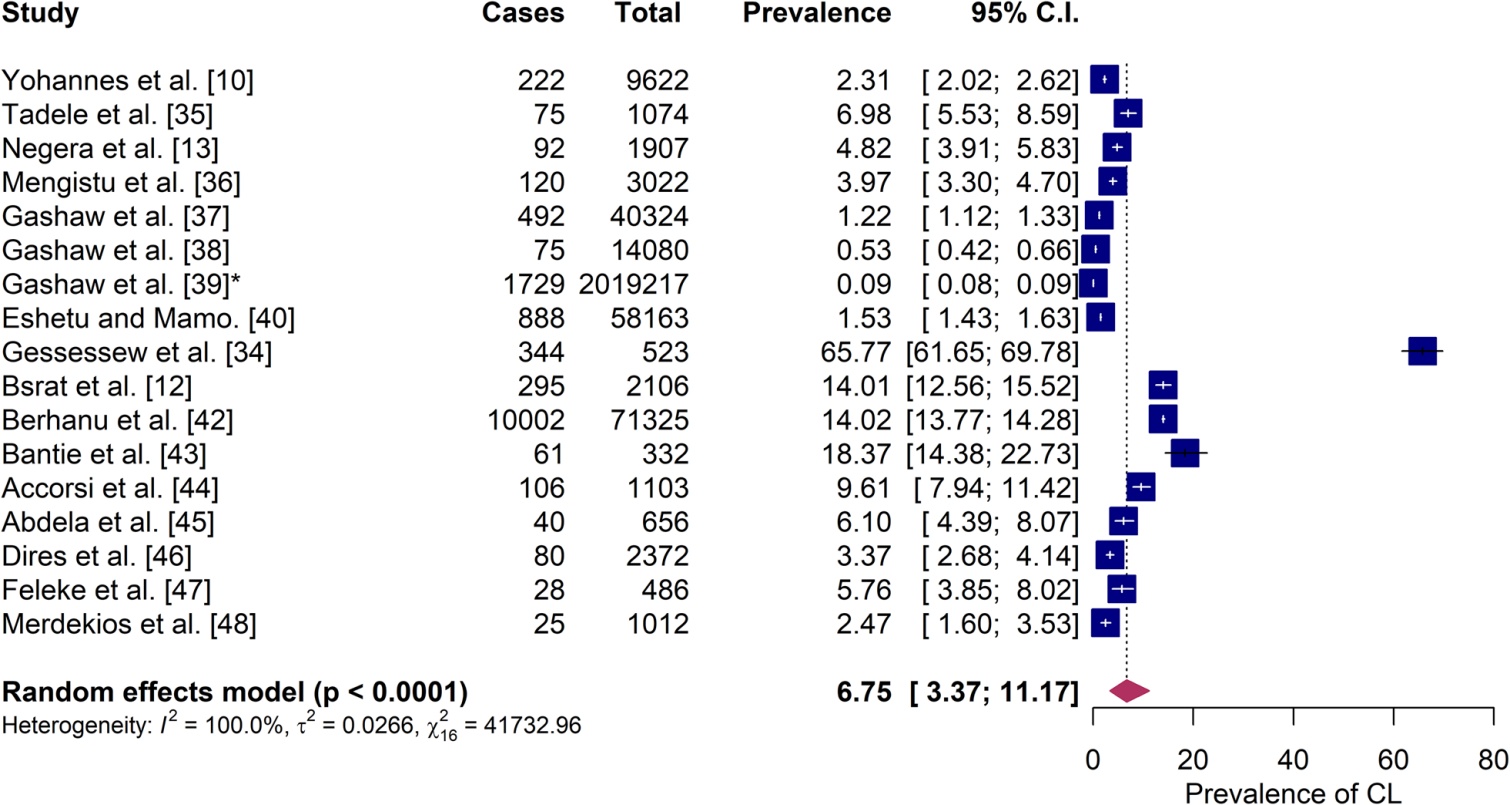
Forest plot showing the prevalence of cutaneous leishmaniasis from 17 studies in Ethiopia

The estimated pooled prevalence of CL in Ethiopia was 6.75% (95% CI 3.37–11.17) on the basis of a random-effects model. Cochran’s *Q* test indicated statistically significant heterogeneity among the included studies (*Q* = 41,732, *P* < 0.001), suggesting that the observed differences in prevalence were unlikely to be due to chance alone. The *I*^2^ statistic was 99.96% (95% CI 99.95–99.99), reflecting that nearly all the observed variability was attributable to true heterogeneity rather than random error. The corresponding *τ*^2^ value was 0.02 (95% CI 0.02–0.10), confirming substantial between-study variation [11–13, 35–48]. (Fig. 2).

Because several studies reported low prevalence estimates (< 0.2), the double arcsine transformation was applied to stabilize the variance before pooling. On the basis of the Studentized residual test, the study by Gessessew Bugssa [35] was identified as an outlier, which was confirmed through visual inspection using leave-one-out diagnostics (Additional file 2: Fig. S1). Excluding this study reduced the pooled prevalence to 4.78%, indicating its influential effect on the overall estimate. Additionally, the forest plot showed that the effect size of this study deviated noticeably from the original pooled prevalence. This deviation highlights the substantial influence of this original study on the overall estimate when included or omitted.

### Subgroup analysis and meta-regression

**Fig. 3:**
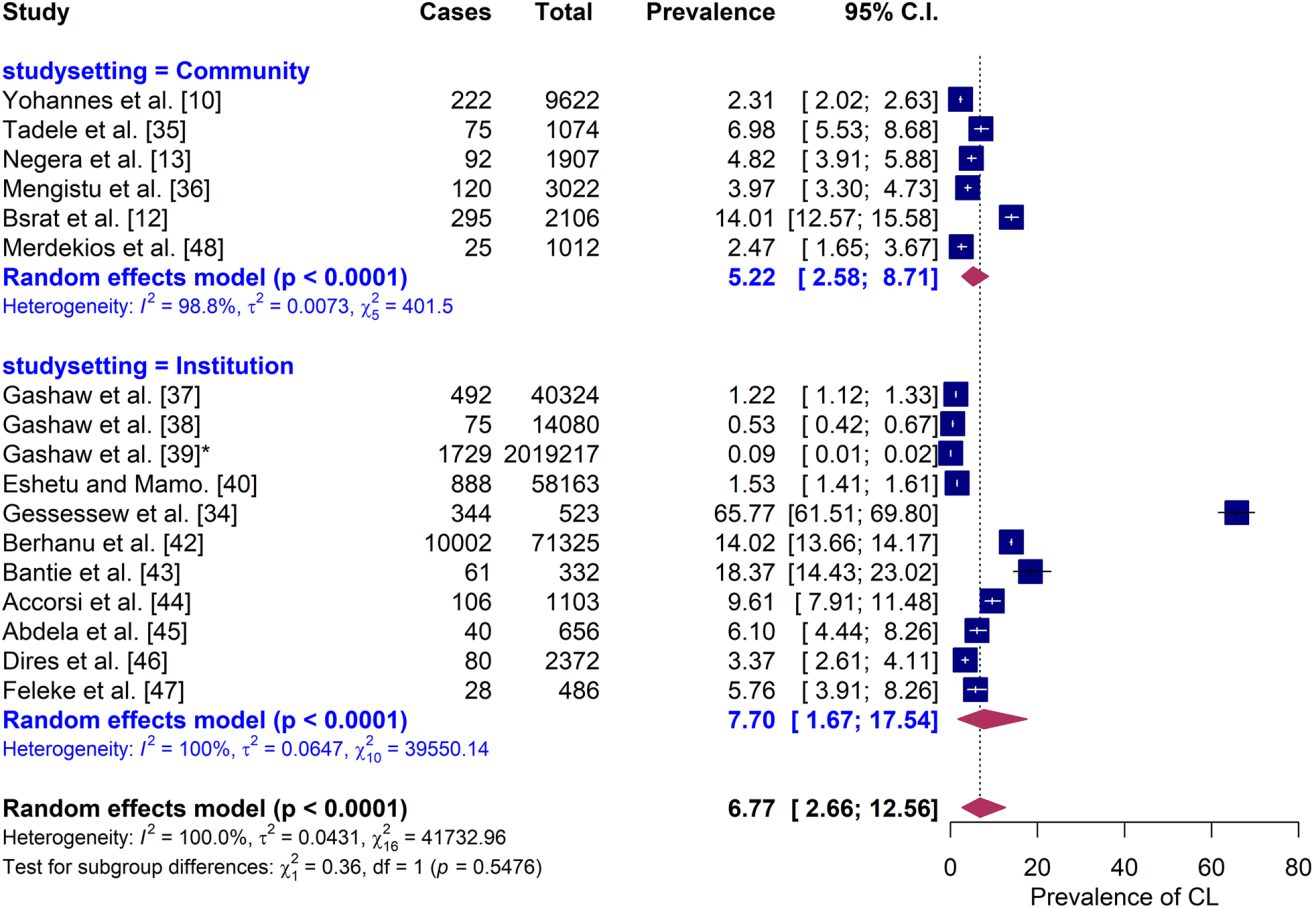
Forest plot of cutaneous leishmaniasis prevalence in Ethiopia, stratified by study setting

Subgroup analysis by study setting showed that institution-based studies had a higher pooled prevalence (7.70%, 95% CI 1.67–17.54%) compared with community-based studies (5.22%, 95% CI 2.58–8.71%) (Fig. 3). However, high heterogeneity persisted in both groups (*I*^2^ = 99–100%), suggesting that study setting alone did not account for the observed variability. No strong evidence of publication bias was detected based on Egger’s regression test (*P* = 0.10) and visual assessment of the funnel plot (Additional file 3: Fig. S2).

### Risk factors of CL

Among the eight included studies, 11 factors had a significant association at 95% CI, which encompassed environmental, household, and individual factors. Notably, individuals living within a 300-m radius of a gorge, a rock, or a cave had a 2.64- and 2.84-times greater likelihood of infection than those living beyond this distance (Table 1).

**Table 1:** Risk factors associated with cutaneous leishmaniasis in Ethiopia: results from a systematic review and meta-analysis, 2025

Determinant	Comparison	Total observation	Pooled OR (95% CI)	*P*-value	Heterogeneity	
					*I*^2^	P-value
Gender	Male	913	3.02 [2.16–4.21]	< 0.001	0.0%	0.50
	Female		Ref			
Age	0–9 versus 10–19	5867	1.58 [1.25–1.99]	0.0001	42.1%	0.1889
	10–19 versus 20–29	4567	3.19 [2.00–5.07]	< 0.001	0.0%	0.8479
	≥ 30	4415	Ref			
Muddy house	Yes	796	1.97 [1.65–2.36]	< 0.001	7.7%	0.2980
	No		Ref			
Cracked walls	Yes	2934	2.36 [1.41–3.95]	0.001	48.1%	0.1650
	No		Ref			
Sleep under a tree/outdoors	Yes	12,059	1.81 [1.48–2.22]	< 0.0001	44.9%	0.1422
	No		Ref			
Gorge near home	Present	12,759	2.64 [1.57–4.44]	0.0002	86.5%	< 0.0001
	Absent		Ref			
Rock/cave near home	Present	10,390	2.84 [2.20–3.67]	< 0.0001	0.0%	0.4824
	Absent		Ref			
Hyrax	Present	13,563	3.65 [2.07–6.42]	< 0.0001	85.9%	< 0.0001
	Absent		Ref			
Living near farmland	Yes	2311	2.60 [1.28–5.28]	0.0081	82.5%	0.0167
	No		Ref			
Animal burrows near home	Present	2520	3.03 [1.56–5.88]	< 0.0001	78.5%	0.0096
	Absent		Ref			
Animal dung near home	Present	3118	1.88 [1.54–2.30]	< 0.0001	2.0%	0.3123
	Absent		Ref			

*Ref* reference category, *OR* odds ratio, *C.I.* confidence interval

### Ecoepidemiology of CL

**Fig. 4:**
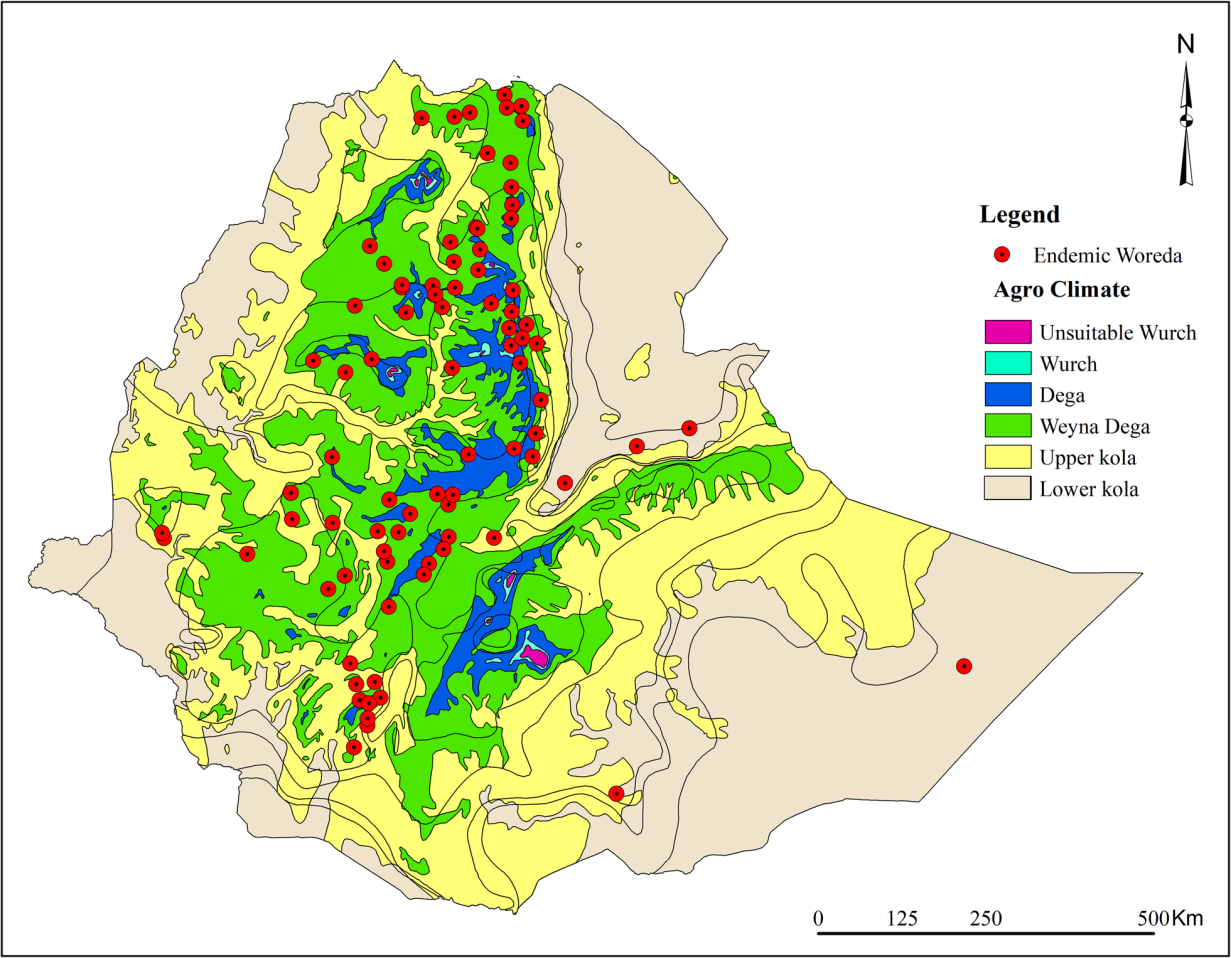
The agro-ecology of cutaneous leishmaniasis endemic woredas in Ethiopia, based on findings from the 2025 systematic review and meta-analysis

CLs is reported in 85 (7.9%) of 1082 woredas, most of which (56%) were in the midland, specifically in the Weyna Dega agroecological zone. Dega, Upper Kola, and Lower Kola contributed 23.5%, 11.8%, and 5.9%, respectively (Fig. 4).

### Ecoepidemiology of sand fly and reservoir hosts

**Fig. 5:**
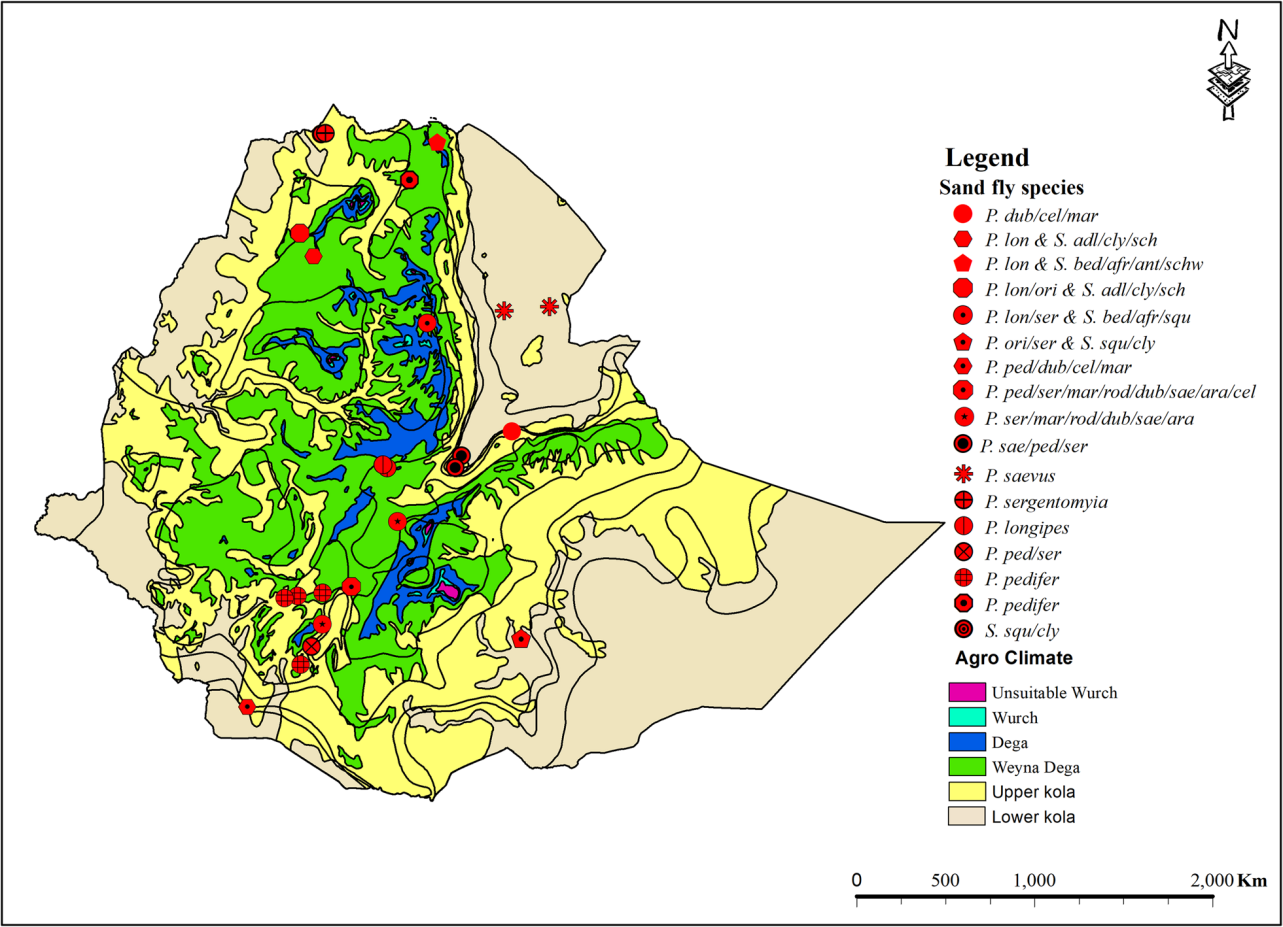
The agro-ecology of incriminated sand fly vectors of cutaneous leishmaniasis in Ethiopia, based on findings from the 2025 systematic review and meta-analysis

**Fig. 6:**
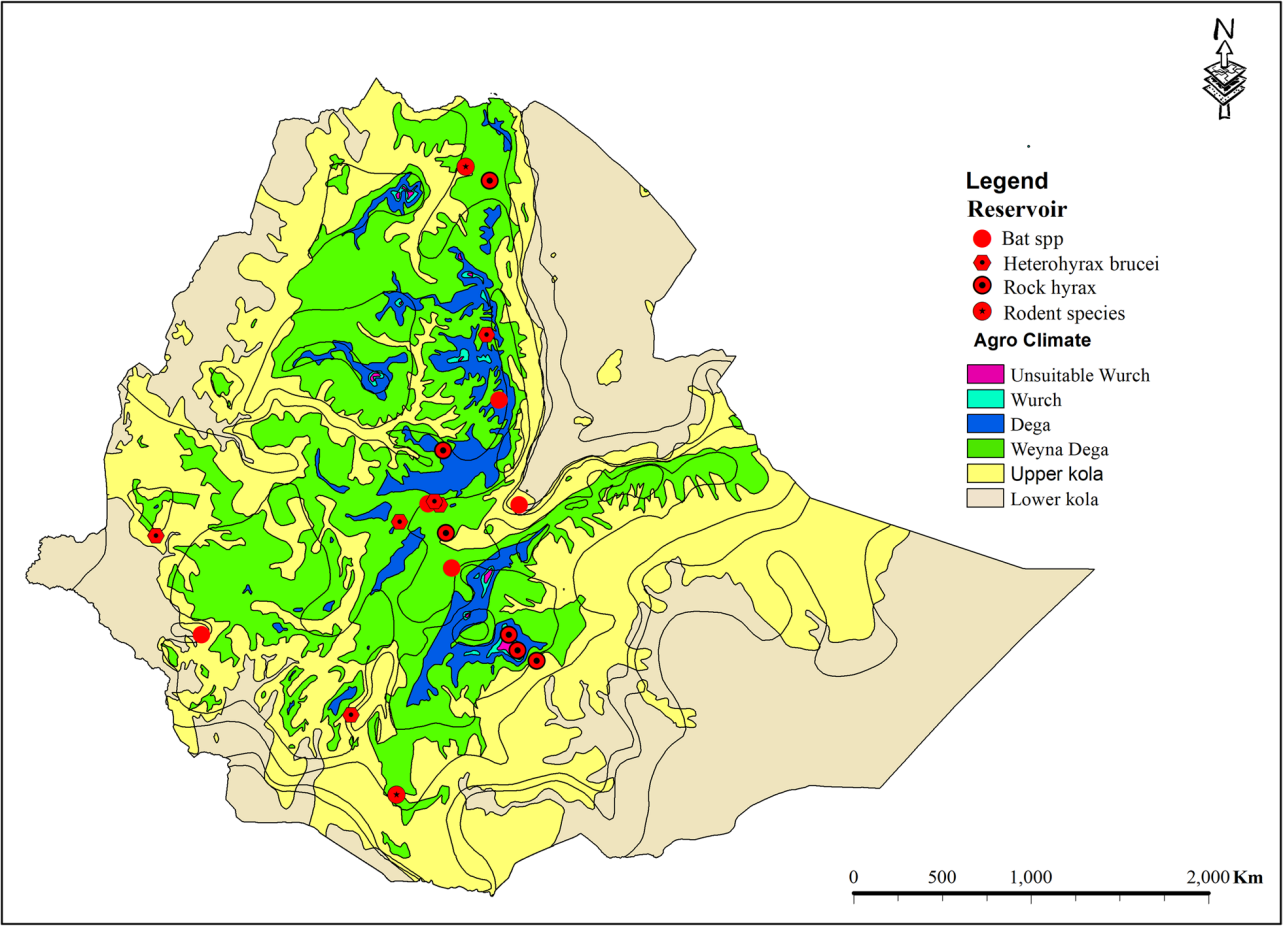
The agro-ecology of reservoir hosts for cutaneous leishmaniasis–causing *Leishmania* species in Ethiopia, based on findings from the 2025 systematic review and meta-analysis

Figures 5 and 6 summarize the vector and reservoir species identified so far. Eleven species of *Phlebotomus* and seven species of *Sergentomyia* have been implicated as vectors for one of the CL-causing species in Ethiopia: *L. aethiopica*, *L. tropica*, and *L. donovani,* as well as *L. major*, which have not been described from humans. Most reports were from the Weyna Dega and the upper Kolla ecological zones. Hyraxes, bats, and rodents were identified as playing a role in the transmission cycle of CL [8, 49–56]. Additional file 1: Table S2 summarizes the identified vector and reservoir species, the corresponding *Leishmania* species, and studies from which these data were obtained.

### Access to care for CL

**Fig. 7:**
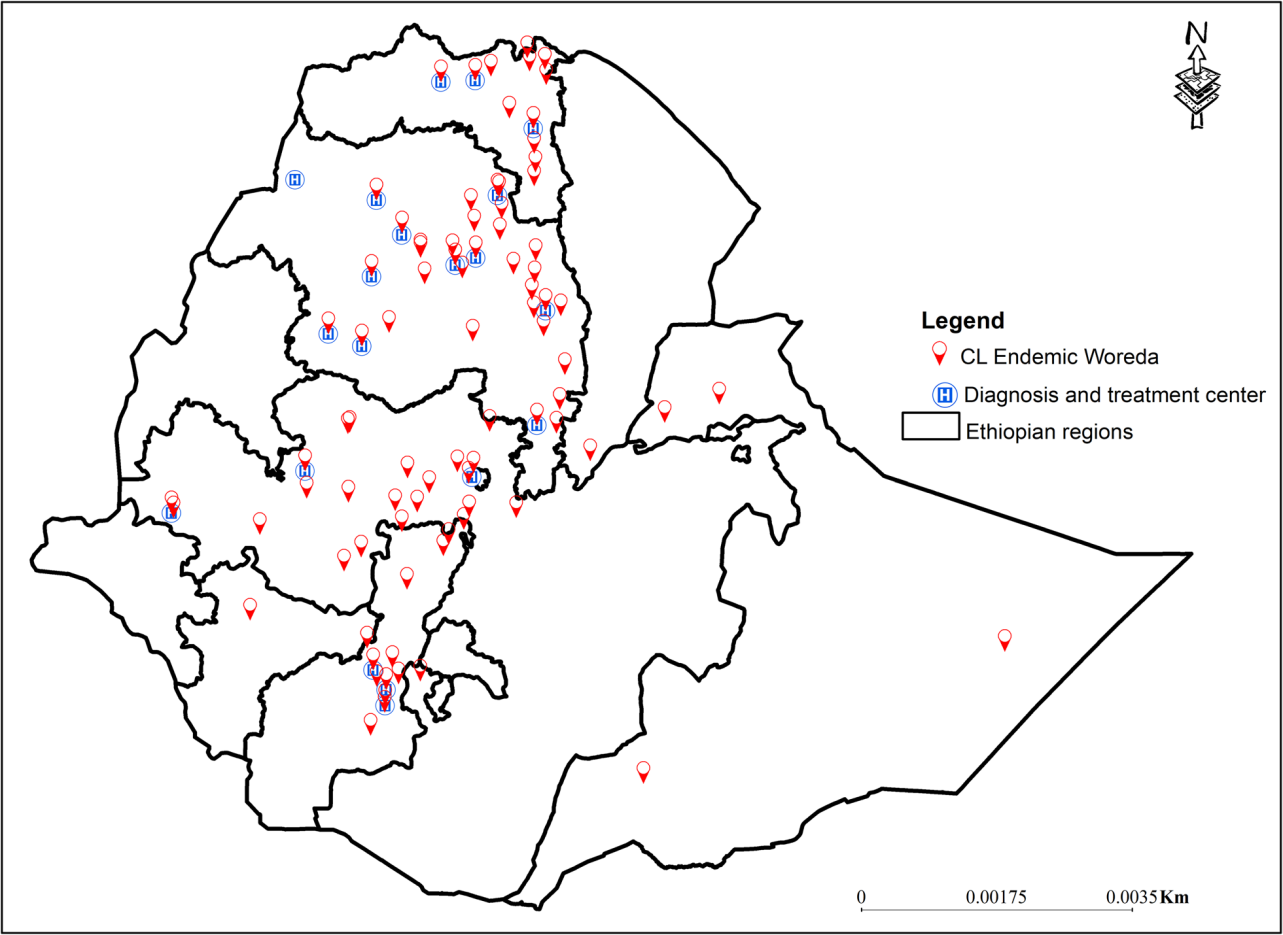
Cutaneous leishmaniasis endemic woredas in Ethiopia (red pins) and the nearest diagnostic and treatment centers, based on data from the 2025 systematic review and meta-analysis

There are 20 diagnosis and treatment centers, as well as the WHO Ethiopia Country Office, serving 85 districts that report CL (Fig. 7).

Over 40% of the districts reporting CL do not have treatment centers at both the woreda and zonal levels. In comparison, about 12% of patients must travel to zonal cities to access diagnosis and treatment services.

### Trend of treatment seeking for CL

**Fig. 8:**
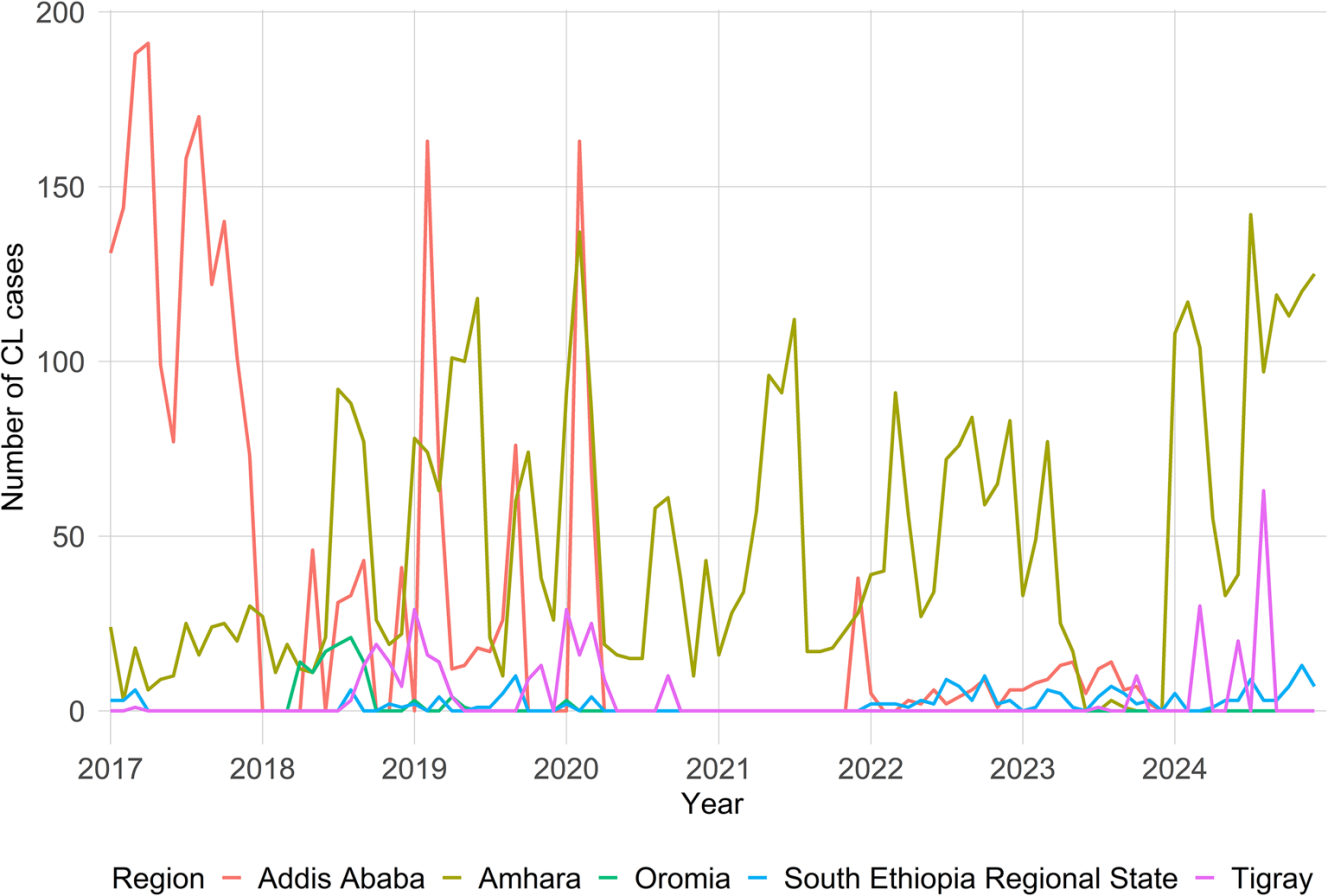
Monthly Trend of cutaneous leishmaniasis across endemic regions of Ethiopia from 2017 to 2024, Summary of the report of WHO-Ethiopia country office

Based on the WHO Ethiopia Country Office data from 2017 to 2024, a total of 7836 CL cases were reported (Additional file 1: Table S3). The largest report was in 2017 (1817 cases), followed by a fluctuating but overall declining trend. By 2023, there were only 346 cases, which increased to 1339 cases by 2024. Also, the proportion varied across regions. The Amhara Regional State consistently reported the highest proportion of the total report, increasing from 17.3% in 2017 to a peak of 93.4% in 2021, and remaining high at 87.5% in 2024. Notably, the region has 11 health facilities providing CL services and 32 endemic woredas. In contrast, Addis Ababa and Oromia showed a declining trend, with Addis Ababa dropping from 82.1% in 2017 to 0% in 2024. Meanwhile, Oromia peaked at 12.3% in 2018 before reporting 0% from 2021 onward, with three facilities available for treating cases from 20 endemic woredas. South Ethiopia, formerly the South Ethiopia Nations and Nationalities Peoples’ Region (SNNPR), and Tigray, followed similar patterns of initially low contributions, a temporary absence of reported cases. Cases in Tigray increased to 8.4% in 2024, having three facilities expected to treat cases from 12 endemic woredas in the region. Meanwhile, in the SNNPR, the case proportion rose to 9.8% of the total national population by 2023, with three health facilities serving 14 endemic woredas (Fig. 8).

## Discussion

The present study assessed the pooled prevalence, temporal patterns, risk factors, and ecoepidemiology of CL in Ethiopia, considering factors such as agroecological zones, the availability of treatment centers, vectors, and reservoir hosts.

The findings showed that the evidence base for CL in Ethiopia remains limited and heterogeneous. Even though there are some studies estimating the burden of CL, some lack specific targets, mixing estimates of Visceral Leishmaniasis (VL) and CL [57], while others reported leishmaniasis in humans and animals [58]. The estimated pooled prevalence, 6.75% (95% CI 3.37–11.17%), in the current study was lower than a study reported by Shita et al., who found an overall pooled prevalence of CL in humans of 20.4% with significant regional differences: 65.9% in the southern part of Ethiopia and 1.53% in the Amhara Regional State [59]. Variations between the current study and the previous review could be attributed to differences in the kind of studies included. Indeed, while the previous study used “clinical CL cases” as the denominator for the facility-based study, we considered “all cases that sought dermatology services” as the numerator. In contrast, the numerator was “parasitologically confirmed” CL in both studies. Also, an SRMA in East Africa [60] came up with a pooled prevalence of 22.57% (95% C.I. 14.36–30.78%) for CL in the region. The major issue with this study, as well, was the high prevalence of institution-based studies included in a SRMA that used “clinical CL cases” instead of “all cases that sought dermatology services” as a denominator.

Household and environmental conditions, along with individual behaviors, contributed to CL prevalence. Both primary studies and published reviews indicate that younger individuals, particularly children under the age of 10 years, are at higher risk of contracting CL. Living with domestic animals, especially goats and sheep, has been consistently linked to an increased risk of CL. Spending time outdoors, especially in the evening and at night when sand flies are most active, is a significant risk factor for CL. This aligns with a study conducted in Kenya, which identified staying outside after sunset as a major risk factor, where individuals who did so were four times more likely to contract CL. Furthermore, previous studies and reviews conducted in Ethiopia emphasized that poor housing conditions, muddy homes, cracked walls, and sleeping outdoors are significant environmental risk factors [57, 59]. Moreover, living near farmland, gorges, or rock/cave, and animal burrows significantly increases the risk of CL, as these conditions provide ideal habitats for sand flies and reservoir hosts [61, 62].

CLs showed a wide ecoepidemiology, with reports covering the Weyna Dega, Dega, and Upper Kola agroecological zones [16, 59]. The number of woredas from where CL was reported was higher in the Weyna Dega and Dega agroecology regions. The predominance of CL cases in the Weyna Dega zone aligns with previous studies from Ethiopia, which have reported higher transmission in midland areas owing to ecological conditions favorable for the sand fly vector [8, 16]. This is because of optimal humidity, moderate temperatures, and the presence of caves and rocky habitats that support both the sand fly vector and hyrax reservoir populations.

Furthermore, vector adaptation and human settlement can be linked to the high burden of CL in the midland (Weyna Dega and Dega) zones. These primary agrarian areas encompass the mountains and plateaus of the eastern and western escarpments of the Ethiopian Rift Valley, characterized by rock crevices, caves, and greater precipitation and vegetation cover, typical habitats for sand flies and hyraxes [62]. Additionally, environmental factor-based risk modeling revealed that slope, elevation, and annual rainfall are effective predictors of CL presence in each area. However, one must exercise caution, as this bias may stem from limited reporting from the lowlands; this is supported by the recent outbreak of CL due to *L. tropica* in military personnel deployed in the unsettled lowlands of the Somali Regional State [19].

The reported CL case load is just the tip of the iceberg. Indeed, it is worth mentioning that, while the estimated annual incidence ranges from 20,000 to 40,000 cases [1], only 1473 cases were officially reported through routine WHO surveillance in 2023–2024. In contrast, a cohort study at ALERT Comprehensive Specialized and Boru Meda General Hospitals enrolled 674 parasitologically confirmed CL patients during the same period [63]. The specific period of gross underreporting shows the weakness of the formal reporting system. The recent years’ reporting could be attributed to the disruption of health services related to the ongoing internal conflict. Overall, the inadequate surveillance, both passive and active, coupled with a prevalent reliance by CL patients on traditional healers, largely due to mistrust of biomedical services, lack of awareness, and the high financial burden of diagnosis and treatment, represent major barriers to accessing allopathic healthcare for CL in Ethiopia [64].

The current evidence reveals persistent gaps in service coverage and marked geographic disparities in the diagnosis and treatment of CL in Ethiopia, which also corroborates the underreporting. Only a few facilities, most in the zonal capital or regional/national referral centers, have significant barriers to access. These findings are consistent with earlier studies [15, 60], which identified the inadequate distribution of CL services, particularly in rural endemic regions. This calls for the need for better accessible, integrated neglected tropical disease (NTD) service delivery at the primary healthcare level, as emphasized in the WHO’s 2030 NTD road map and the third “2021 to 2025” national NTD master plan [64]. Furthermore, these findings indicate that the main risk factors for CL in Ethiopia, such as being male, younger age, poor housing, and living close to caves, gorges, or animal habitats, are also common in many African settings. They reflect how rural lifestyles, farming activities, and housing conditions across the continent increase people’s contact with sand flies and reservoir animals [10, 65, 66]. This suggests that prevention efforts in Africa should focus on improving housing, reducing breeding sites near homes, and raising awareness among high-risk groups, especially young men in farming communities.

The current study provides comprehensive data on the prevalence, risk factors, and spatial distribution of CL, offering valuable insights for tailoring control and elimination efforts. However, there is a lack of sufficient articles related to disease prevalence, reservoir hosts, and sand fly vectors, which limits deeper understanding of the transmission of the disease and risk factors. Furthermore, most articles are health institution-based, which may not fully capture the situation of rural underserved communities, potentially overlooking a broader range of cases.

## Conclusions

The estimated pooled prevalence may underestimate the true burden of disease. The identified risk factors were more related to rural livelihoods and living conditions, and most of the endemic woredas were in the most productive agrarian agroecological zones, which underlines the health and socioeconomic significance of CL in Ethiopia. The limited access to care might contribute to the perpetuation of the challenge. There is also a lack of entomological data on vectors in these areas. Thus, decentralizing healthcare services and improving surveillance for CL are crucial steps in breaking the vicious cycle of poverty.

## Supplementary Information

**Additional file 1: Table S1.** Output for test screening for externally Studentized residuals. **Table S2.** *Leishmania* Species, sand fly vectors, and reservoir hosts reported from cutaneous leishmaniasis endemic areas in Ethiopia, 2025. **Table S3.** Number of confirmed cutaneous leishmaniasis cases treated across the treatment centers in Ethiopia from 2017 to 2024, WHO.

**Additional file 2:** **Figure S1.** Funnel plot for assessing publication bias in the meta-analysis of proportions for the prevalence of cutaneous leishmaniasis in Ethiopia, 2025.

**Additional file 3:** **Figure S2.** Leave-one-out forest plot to visually inspect the influence of outlier studies on the pooled proportion, for the prevalence of cutaneous leishmaniasis in Ethiopia, 2025.

## Data Availability

Data supporting the main conclusions of this study are included in the manuscript.

## Abbreviations

CI: Confidence interval
CL: Cutaneous leishmaniasis
OR: Odds ratio
NTD: Neglected tropical disease
PRISMA: Preferred Reporting Items for Systematic Reviews and Meta-Analyses
SRMA: Systematic review and meta-analysis
WHO: World Health Organization
NNPR: South Ethiopia Nations and Nationalities Peoples’ Region

## References

[1] World Health Organization. Leishmaniasis: fact sheet. Geneva: World Health Organization; 2023. https://www.who.int/news-room/fact-sheets/detail/leishmaniasis. Accessed 16 Jan 2026.

[2] Reithinger R, Dujardin JC, Louzir H, Pirmez C, Alexander B, Brooker S. Cutaneous leishmaniasis. Lancet Infect Dis. 2007;7:581–96. 10.1016/S1473-3099(07)70209-8. 17714672 10.1016/S1473-3099(07)70209-8

[3] Karimkhani C, Wanga V, Coffeng LE, Naghavi P, Dellavalle RP, Naghavi M. Global burden of cutaneous leishmaniasis: a cross-sectional analysis from the Global Burden of Disease Study 2013. Lancet Infect Dis. 2016;16:584–91. 10.1016/S1473-3099(16)00003-7. 26879176 10.1016/S1473-3099(16)00003-7

[4] World Health Organization, Regional Office for the Eastern Mediterranean. Manual for case management of cutaneous leishmaniasis in the WHO Eastern Mediterranean Region. Cairo: WHO Regional Office for the Eastern Mediterranean; 2013. https://apps.who.int/iris/handle/10665/120002. Accessed 16 Jan 2026.

[5] World Health Organization. Leishmaniasis: fact sheet. Geneva: World Health Organization; 2022. https://www.who.int/news-room/fact-sheets/detail/leishmaniasis. Accessed 16 Jan 2026.

[6] Bamorovat M, Sharifi I, Agha Kuchak Afshari S, Ghasemi Nejad Almani P. Mutual role of patients and the healthcare system in the control of cutaneous leishmaniasis. Transbound Emerg Dis. 2023;70:7814940. 10.1155/2023/7814940. 10.1155/2023/7814940 PMC12016944 40303822

[7] Alvar J, Vélez ID, Bern C, Herrero M, Desjeux P, Cano J, et al. Leishmaniasis worldwide and global estimates of its incidence. PLoS ONE. 2012;7:e35671. 10.1371/journal.pone.0035671. 22693548 10.1371/journal.pone.0035671 PMC3365071

[8] Ashford RW, Bray MA, Hutchinson MP, Bray RS. The epidemiology of cutaneous leishmaniasis in Ethiopia. Trans R Soc Trop Med Hyg. 1973;67:568–601. 10.1016/0035-9203(73)90088-6. 4150462 10.1016/0035-9203(73)90088-6

[9] Lemma A, Foster WA, Gemetchu T, Preston PM, Bryceson A, Minter DM. Studies on leishmaniasis in Ethiopia: I: preliminary investigations into the epidemiology of cutaneous leishmaniasis in the highlands. Ann Trop Med Parasitol. 1969;63:455–72. 10.1080/00034983.1969.11686649. 5394018

[10] Yohannes M, Abebe Z, Boelee E. Prevalence and environmental determinants of cutaneous leishmaniasis in rural communities in Tigray, northern Ethiopia. PLoS Negl Trop Dis. 2019;13:e0007722. 10.1371/journal.pntd.0007722. 31557152 10.1371/journal.pntd.0007722 PMC6782111

[11] Seid A, Gadisa E, Tsegaw T, Abera A, Teshome A, Mulugeta A, et al. Risk map for cutaneous leishmaniasis in Ethiopia based on environmental factors as revealed by geographical information systems and statistics. Geospat Health. 2014;8:377. 10.4081/gh.2014.27. 24893015 10.4081/gh.2014.27

[12] Bsrat A, Berhe N, Balkew M, Yohannes M, Teklu T, Gadisa E, et al. Epidemiological study of cutaneous leishmaniasis in Saesie Tsaeda-Emba district, eastern Tigray, northern Ethiopia. Parasit Vectors. 2015;8:149. 10.1186/s13071-015-0758-1. 25889827 10.1186/s13071-015-0758-9 PMC4359476

[13] Lemma W, Erenso G, Gadisa E, Balkew M, Gebre-Michael T, Hailu A. A zoonotic focus of cutaneous leishmaniasis in Addis Ababa, Ethiopia. Parasit Vectors. 2009;2:60. 10.1186/1756-3305-2-60. 19954530 10.1186/1756-3305-2-60 PMC2794267

[14] Negera E, Gadisa E, Yamuah L, Engers H, Hussein J, Kuru T, et al. Outbreak of cutaneous leishmaniasis in Silti woreda, Ethiopia: risk factor assessment and causative agent identification. Trans R Soc Trop Med Hyg. 2008;102:883–90. 10.1016/j.trstmh.2008.03.021. 18479722 10.1016/j.trstmh.2008.03.021

[15] Tegegne B, Yimer M, Geyesus T, Ayal A, Yimer M. Short reports on cutaneous leishmaniasis outbreak investigation in Ankesha-Guagsa district, Amhara region, Northwest Ethiopia. Trop Doct. 2021;52:131–3. 10.1177/00494755211055252. 34894873 10.1177/00494755211055252

[16] Bray RS, Ashford RW, Bray MA. The parasite causing leishmaniasis in Ethiopia. Trans R Soc Trop Med Hyg. 1973;67:345–8. 10.1016/0035-9203(73)90111-9. 4778189 10.1016/0035-9203(73)90111-9

[17] Amare GA, Mekonnen GG, Kassa M, Addisu A, Kendie DA, Tegegne B, et al. First report of cutaneous leishmaniasis caused by *Leishmania donovani* in Ethiopia. Parasit Vectors. 2023;16:457. 10.1186/s13071-023-06057-9. 38104111 10.1186/s13071-023-06057-9 PMC10725588

[18] Hailu A, Di Muccio T, Abebe T, Hunegnaw M, Kager PA, Gramiccia M. Isolation of *Leishmania tropica* from an Ethiopian cutaneous leishmaniasis patient. Trans R Soc Trop Med Hyg. 2006;100:53–8. 10.1016/j.trstmh.2005.04.017. 16154167 10.1016/j.trstmh.2005.04.017

[19] Abera A, Tadesse H, Beyene D, Geleta D, Abose E, Kinde S, et al. Outbreak of cutaneous leishmaniasis caused by Leishmania tropica amongst militia members in a non-endemic district under conflict in the lowlands of Somali Region in Ethiopia [preprint]. medRxiv. 2024. 10.1101/2024.10.05.24314933

[20] Kassahun A, Sadlova J, Dvorak V, Kostalova T, Rohousova I, Frynta D, et al. Detection of *Leishmania donovani* and *L. tropica* in Ethiopian wild rodents. Acta Trop. 2015;145:39–44. 10.1016/j.actatropica.2015.02.006. 25700710 10.1016/j.actatropica.2015.02.006

[21] Kassahun A, Sadlova J, Benda P, Kostalova T, Warburg A, Hailu A, et al. Natural infection of bats with *Leishmania* in Ethiopia. Acta Trop. 2015;150:166–70. 10.1016/j.actatropica.2015.07.024. 26232657 10.1016/j.actatropica.2015.07.024

[22] Ashford RW. A possible reservoir for *Leishmania tropica* in Ethiopia. Trans R Soc Trop Med Hyg. 1970;64:749–50. 10.1016/0035-9203(70)90117-3. 10.1016/0035-9203(70)90117-3 5531392

[23] Killick-Kendrick R. The biology and control of phlebotomine sand flies. Clin Dermatol. 1999;17:279–89. 10.1016/S0738-081X(99)00046-2. 10384867 10.1016/s0738-081x(99)00046-2

[24] Pareyn M, Van den Bosch E, Girma N, van Houtte N, Van Dongen S, Van der Auwera G, et al. Ecology and seasonality of sandflies and potential reservoirs of cutaneous leishmaniasis in Ochollo, a hotspot in southern Ethiopia. PLoS Negl Trop Dis. 2019;13:e0007667. 10.1371/journal.pntd.0007667. 31425506 10.1371/journal.pntd.0007667 PMC6715250

[25] Chelbi I, Abdi A, Depaquit J, Fares W, Abbas MA, Dachraoui K, et al. Investigation of the sandfly fauna of central arid areas and northern humid regions of Tunisia, with morphological and molecular identification of *Phlebotomus* (*Larroussius*) *perfiliewi*. Insects. 2022;13:1057. 10.3390/insects13111057. 36421960 10.3390/insects13111057 PMC9696294

[26] Korine C, Niv A, Axelrod M, Dahan T. Species richness and activity of insectivorous bats in cotton fields in semi-arid and mesic Mediterranean agroecosystems. Mamm Biol. 2020;100:73–80. 10.1007/s42991-019-00002-z.

[27] Asefa A, Kebede T, Tadesse D, Fikre H, Abate M, Mengistu G, et al. Eco-epidemiology of cutaneous leishmaniasis in Ethiopia: a systematic review and meta-analysis [protocol]. PROSPERO; 2024. https://www.crd.york.ac.uk/PROSPERO/view/CRD42024571850. Accessed 16 Jan 2026.

[28] Page MJ, McKenzie JE, Bossuyt PM, Boutron I, Hoffmann TC, Mulrow CD, et al. The PRISMA 2020 statement: an updated guideline for reporting systematic reviews. BMJ. 2021;372:n71. 10.1136/bmj.n71. 33782057 10.1136/bmj.n71 PMC8005924

[29] Central Statistical Agency of Ethiopia. 2007 population and housing census of Ethiopia: administrative report. Addis Ababa: Central Statistical Agency of Ethiopia; 2010. https://www.ethiopianreview.com/pdf/001/Cen2007_firstdraft(1).pdf. Accessed 16 Jan 2026.

[30] Duguma B, Janssens GP. Assessment of livestock feed resources and coping strategies with dry season feed scarcity in mixed crop–livestock farming systems around the Gilgel Gibe catchment, southwest Ethiopia. Sustainability. 2021;13:10713. 10.3390/su131910713.

[31] Mesfin D, Assefa E, Simane B. Variability of soil quality indicators along different landscape positions of the Choke Mountain agroecosystem, upper Blue Nile Basin, Ethiopia. Heliyon. 2022;8:e09850. 10.1016/j.heliyon.2022.e09850. 35815145 10.1016/j.heliyon.2022.e09850 PMC9264026

[32] Simane B, Zaitchik BF, Ozdogan M. Agroecosystem analysis of the Choke Mountain watersheds, Ethiopia. Sustainability. 2013;5:592–616. 10.3390/su5020592.

[33] Mesfin D, Assefa E, Simane B. Characteristics of soil quality attributes under different agroecosystems and its implications for agriculture in the Choke Mountain watershed in Ethiopia. Front Agric Sci Eng. 2024. 10.15302/J-FASE-2023502.

[34] Federal Democratic Republic of Ethiopia, Ministry of Health. Environmental and Social Management Framework (ESMF) for Additional Financing for Ethiopia COVID-19 Emergency Response Project (P173750)—Revised Report. World Bank; 2021. https://documents1.worldbank.org/curated/en/578831623327575178/pdf/ESMF-Ethiopia-COVID-19.pdf.

[35] Bugssa G, Hailu A, Demtsu B. The current status of cutaneous leishmaniasis and the pattern of lesions in Ochollo primary school students, Ochollo, Southwestern Ethiopia. Sci J Clin Med. 2014;3:111. 10.11648/j.sjcm.20140306.13.

[36] Tadele G, Samuel A, Fite MB, Tasew G, Abera A. Prevalence and knowledge of cutaneous leishmaniasis in Aleku area of Sayo District, Western Ethiopia: a community-based cross-sectional study. J Med. 2024;5:1107.

[37] Mengistu G, Laskay T, Gemetchu T, Humber D, Ersamo M, Evans D, et al. Cutaneous leishmaniasis in south-western Ethiopia: Ochollo revisited. Trans R Soc Trop Med Hyg. 1992;86:149–53. 10.1016/0035-9203(92)90546-O. 1440773 10.1016/0035-9203(92)90546-o

[38] Gashaw B, Yizengaw E, Sebsibe B, Mulu B, Alemu T, Nibret E. Clinical manifestations and traditional practice in patients with cutaneous leishmaniasis: do leishmaniasis induce high blood glucose levels? Ethiop J Health Dev. 2025;37:1–10. https://www.ajol.info/index.php/ejhd/article/view/291109

[39] Gashaw B, Yizengaw E, Yismaw G, Tebeje S, Tilahun F, Sebsibe B, et al. Cutaneous leishmaniasis: its burden and challenge for patients and the health care system at Boru Meda Hospital, North-Central Ethiopia. Ethiop J Health Dev. 2023;37:123–30.

[40] Gashaw B, Yizengaw E, Nibret E, Workineh A, Abebe A. Epidemiological and clinical profiles of cutaneous leishmaniasis cases in Amhara National Regional State, Northwest Ethiopia: a multicenter retrospective study. Dermatol Rep. 2024;17:10089. 10.4081/dr.2024.10089. 10.4081/dr.2024.10089 PMC11863562 39912729

[41] Eshetu B, Mamo H. Cutaneous leishmaniasis in north-central Ethiopia: trend, clinical forms, geographic distribution, and determinants. Trop Med Health. 2020;48:39. 10.1186/s41182-020-00231-w. 32518497 10.1186/s41182-020-00231-w PMC7271444

[42] Berhanu A, Dugassa S, Maru M, Animut A, Erko B, Hailu A, et al. Cutaneous leishmaniasis in Kutaber District, Ethiopia: prevalence, sand fly fauna and community knowledge, attitude and practices. Heliyon. 2023;9:e18286. 10.1016/j.heliyon.2023.e18286. 37520994 10.1016/j.heliyon.2023.e18286 PMC10382297

[43] Bantie B, Kassaw G, Demelash AT, Abate MW, Nigat AB, Amare AT, et al. Magnitude and associated factors of cutaneous leishmaniasis among patients visiting Nefas Mewcha Primary Hospital, Northern Ethiopia, 2022: an institution-based cross-sectional study. BMJ Open. 2024;14:e075549. 10.1136/bmjopen-2023-075549. 38176880 10.1136/bmjopen-2023-075549 PMC10773395

[44] Accorsi S, Barnabas GA, Farese P, Padovese V, Terranova M, Racalbuto V, et al. Skin disorders and disease profile of poverty: analysis of medical records in Tigray, northern Ethiopia, 2005–2007. Trans R Soc Trop Med Hyg. 2009;103:469–75. 10.1016/j.trstmh.2008.11.028. 19136130 10.1016/j.trstmh.2008.11.028

[45] Abdela SG, Diro E, Zewdu FT, Berhe FT, Yeshaneh WE, Tamirat KS, Tweya H, Timire C, Van Griensven J. Looking for NTDs in the skin; an entry door for offering patient centered holistic care. J Infect Dev Ctries. 2020;14(6.1):165–215. 10.3855/jidc.11707. 10.3855/jidc.11707 32614791

[46] Dires A, Gedamu S, Kumar P, Yimam W, Ademe S, Dires T. Determinants of cutaneous leishmaniasis among students in Delanta district, northeast Ethiopia: a case–control study. Health Sci Rep. 2022;5:e917. 10.1002/hsr2.917. 36324427 10.1002/hsr2.917 PMC9621467

[47] Tilahun F, Alemu W, Mulatu G. Magnitude and associated factors of cutaneous leishmaniasis in Mekelle city, Ayder Referral Hospital, Tigray, northern Ethiopia, 2014. Clin Med Res. 2014;3:189–99. 10.11648/j.cmr.20140306.16.

[48] Merdekios B, Kote M, Pareyn M, Van Geertruyden JP, van Griensven J. Prevalence and risk factors of cutaneous leishmaniasis in a newly identified endemic site in south Ethiopia. PLoS ONE. 2024;19:e0311917. 10.1371/journal.pone.0311917. 39775255 10.1371/journal.pone.0311917 PMC11684615

[49] Alemayehu B, Tomas T, Koroto N, Matusala T, Megaze A, Leirs H. Abundance and distribution of *Phlebotomus pedifer* (Diptera: Psychodidae) across various habitat types in endemic foci of cutaneous leishmaniasis in southern Ethiopia. Trop Med Infect Dis. 2024;9:302. 10.3390/tropicalmed9120302. 39728829 10.3390/tropicalmed9120302 PMC11679830

[50] Jemberie W, Animut A, Dugassa S, Gebresilassie A, Melkamu R, Aklilu E, et al. Ecology and infection status of sand flies in rural and urban cutaneous leishmaniasis endemic areas in northwest Ethiopia. Trop Med Infect Dis. 2024;9:52. 10.3390/tropicalmed9030052. 38535875 10.3390/tropicalmed9030052 PMC10974852

[51] Balkew M, Gebre-Michael T, Berhe N, Ali A, Hailu A. Leishmaniasis in the middle course of the Ethiopian Rift Valley: II. entomological observations. Ethiop Med J. 2002;40:271–82. 12602251

[52] Gebre‑Michael T, Balkew M, Ali A, Ludovisi A, Gramiccia M. The isolation of *Leishmania tropica* and *L. aethiopica* from *Phlebotomus* (*Paraphlebotomus*) species (*Diptera*: *Psychodidae*) in the Awash Valley, northeastern Ethiopia. Trans R Soc Trop Med Hyg. 2004;98:64–70. 10.1016/s0035-9203(03)00008-7. 14702839 10.1016/s0035-9203(03)00008-7

[53] Abebe A, Evans DA, Gemetchu T. The isolation of *Leishmania aethiopica* from the ground squirrel *Xerus rutilus*. Trans R Soc Trop Med Hyg. 1990;84:691. 10.1016/0035-9203(90)90147-7. 2278071 10.1016/0035-9203(90)90147-7

[54] Gebre-Michael T, Pratlong F, Lane RP. Phlebotomus (*Phlebotomus*) *duboscqi* naturally infected with *Leishmania major* in southern Ethiopia. Trans R Soc Trop Med Hyg. 1993;87:10–1. 10.1016/0035-9203(93)90399-B. 8465376 10.1016/0035-9203(93)90399-b

[55] Haftom M, Petrucka P, Gemechu K, Nesro J, Amare E, Hailu T, et al. Prevalence and risk factors of human leishmaniasis in Ethiopia: a systematic review and meta-analysis. Infect Dis Ther. 2021;10:47–60. 10.1007/s40121-020-00361-y. 33170497 10.1007/s40121-020-00361-y PMC7652913

[56] Assefa A. Leishmaniasis in Ethiopia: a systematic review and meta-analysis of prevalence in animals and humans. Heliyon. 2018;4:e00723. 10.1016/j.heliyon.2018.e00723. 30101202 10.1016/j.heliyon.2018.e00723 PMC6082994

[57] Al-Ashwal MA, Atroosh WM, Al-Adhroey AH, Al-Subbary AA, Yee-Ling L, Al-Mekhlafi HM. A disfiguring neglected tropical disease sweeps war-torn Yemen. Trans R Soc Trop Med Hyg. 2023;117:823–38. 10.1093/trstmh/trad044. 37486252 10.1093/trstmh/trad044

[58] Ngere I, Gufu Boru W, Isack A, Muiruri J, Obonyo M, Matendechero S, et al. Burden and risk factors of cutaneous leishmaniasis in a peri-urban settlement in Kenya. PLoS ONE. 2020;15:e0227697. 10.1371/journal.pone.0227697. 31971945 10.1371/journal.pone.0227697 PMC6977748

[59] Shita EY, Nibret E, Munshea A, Gashaw B. Burden and risk factors of cutaneous leishmaniasis in Ethiopia: a systematic review and meta-analysis. Int J Dermatol. 2022;61:1336–45. 10.1111/ijd.16265. 35569096 10.1111/ijd.16265

[60] Sarmadi M, Bagherian Z, Ahmadi-Soleimani SM, Rezaiemanesh MR, Khodamoradi F, Rahimi S, et al. Environmental health risk factors and cutaneous leishmaniasis in northeastern Iran. J Vector Borne Dis. 2023;60:372–81. 10.4103/0972-9062.374236. 38174514 10.4103/0972-9062.374236

[61] van Henten S, Adriaensen W, Fikre H, Akuffo H, Diro E, Hailu A, et al. Cutaneous leishmaniasis due to *Leishmania aethiopica*. EClinicalMedicine. 2018;6:69–81. 10.1016/j.eclinm.2018.12.009. 31193672 10.1016/j.eclinm.2018.12.009 PMC6537575

[62] Aklilu E, Yared S, Gebresilassie A, Legesse B, Hailu A. Phlebotomine sandflies (Diptera: Psychodidae) of Ethiopia. Heliyon. 2023;9:e14344. 10.1016/j.heliyon.2023.e14344. 36925525 10.1016/j.heliyon.2023.e14344 PMC10011004

[63] Doni SN, Mohammed FS, Mohammed AB, Zewdu FT, Hailemichael Y, Mosweu I, et al. Clinical and patient-reported outcomes of cutaneous leishmaniasis treatment in Ethiopia: a prospective observational cohort study in two referral hospitals. Br J Dermatol. 2026. 10.1093/bjd/ljag061. 41701626 10.1093/bjd/ljag061

[64] Ministry of Health, Ethiopia. The Third National Neglected Tropical Diseases Strategic Plan 2021–2025 (2013/14–2017/18 E.C.). Addis Ababa: Ministry of Health; 2021. https://www.moh.gov.et/ntd-strategic-plan-2021-2025.pdf. Accessed 16 Jan 2026.

[65] Doni S, Yeneneh K, Hailemichael Y, Gebremichael M, Skarbek S, Ayele S, et al. Health-related quality of life of adults with cutaneous leishmaniasis at ALERT Hospital, Ethiopia. PLoS Negl Trop Dis. 2023;17:e0011196. 10.1371/journal.pntd.0011196. 37903149 10.1371/journal.pntd.0011196 PMC10645367

[66] Kaba M, Hailemichael Y, Alemu AY, Cherkose T, Kebebew G, Kassa FA, et al. Understanding experiences of neglected tropical diseases of the skin in Ethiopia. BMJ Glob Health. 2025;10:e016650. 10.1136/bmjgh-2024-016650. 39914875 10.1136/bmjgh-2024-016650 PMC11800212

