# Supplementary material for: The ecoepidemiology of cutaneous leishmaniasis in Ethiopia: a systematic review and meta-analysis
Source: Parasit Vectors. 2026 May 9;19:241. doi: 10.1186/s13071-026-07376-3 (PMC13238102; doi:10.1186/s13071-026-07376-3)
Supplement: Supplementary file 1 — Additional file 1: Table S1. Output for test screening for externally Studentized residuals. Table S2. Leishmania Species, sand fly vectors, and reservoir hosts reported from cutaneous leishmaniasis endemic areas in Ethiopia, 2025. Table S3. Number of confirmed cutaneous leishmaniasis cases treated across the treatment centers in Ethiopia from 2017 to 2024, WHO. [file 13071_2026_7376_MOESM1_ESM.docx]

**Supplementary Table 1**: Output for test screening for externally studentized residuals

| Study | resid | SE | z |
| --- | --- | --- | --- |
| Gessessew B et al. 2024 | 0.7243 | 0.1663 | 4.3542 |
| Gashaw et al. 2024 | -0.2488 | 0.1566 | -1.5889 |
| Berhanu et al. 2023 | 0.1303 | 0.0916 | 1.4225 |
| Gashaw et al. 2023 | -0.2022 | 0.1739 | -1.1626 |
| Bantie B et al. 2023 | 0.1918 | 0.1701 | 1.1279 |
| Gashaw et al. 2023 | -0.1625 | 0.185 | -0.8781 |
| Ehetu and Mamo 2020 | -0.1485 | 0.1934 | -0.768 |
| Bisrat et al. 2015 | 0.128 | 0.1674 | 0.7645 |
| Yohannes et al. 2019 | -0.1178 | 0.1712 | -0.688 |
| Merdekios et al. 2024 | -0.1106 | 0.1692 | -0.6539 |
| Direst et al. 2021 | -0.0831 | 0.169 | -0.4918 |
| Mengistu et al. 1992 | -0.0664 | 0.169 | -0.3927 |
| Accorsi et al. 2009 | 0.0556 | 0.1686 | 0.33 |
| 1. Negera et al. 2008 | -0.044 | 0.1688 | -0.2609 |
| Feleke T et al. 2014 | -0.0203 | 0.1697 | -0.1196 |
| Abdela et al. 2020 | -0.0133 | 0.1693 | -0.0787 |
| Tadele et al. 2024 | 0.0051 | 0.1688 | 0.03 |

**Supplementary Table 2**: *Leishmania* Species, Sandfly Vectors, and Reservoir hosts reported from cutaneous leishmaniasis endemic areas in Ethiopia, a result from systematic review and meta-analysis, 2025.

.

| **SN** | **CL vectors and reservoirs** | **References** |
| --- | --- | --- |
| 1 | ***Leishmania aethiopica*** |  |
|  | Vectors |  |
|  | *Phlebotomus pedifer* | [8, 34] |
|  | *Phlebotomus longipes* | [8, 35, 36] |
|  | *Phlebotomus celiae* | [36] |
|  | *Phlebotomus martini* | [36] |
|  | *Phlebotomus rodhaini* | [36] |
|  | *Phlebotomus duboscqui* | [36] |
|  | *Phlebotomus arabicus* | [36] |
|  | *Paraphlebotomus saevus* | [36, 37] |
|  | *Paraphlebotomus sergenti* | [36, 37] |
|  | Reservoir host |  |
|  | *Heterohyrax brucei* | [8, 38] |
|  | *Xerus rutilus* | [39] |
|  | *Procavia habessinica* | [8] |
| 2 | ***Leishmania tropica*** |  |
|  | Vectors |  |
|  | *Paraphlebotomus sergenti* | [37] |
|  | *Paraphlebotomus saevus* | [37] |
|  | Reservoir host |  |
|  | Rodent (Acomys spp, Arvicanthis spp, Gerbillus spp.) | [40] |
|  | Bat (Cardioderma cor) | [41] |
| 3 | ***Leishmania major*** |  |
|  | Vector |  |
|  | *Phlebotomus duboscqui* | [42] |
|  | Reservoir host |  |
|  | Bat (Nycteris hispida) | [41] |
| 4 | ***Leishmania donovani*** |  |
|  | Vector* |  |
|  | Reservoir host |  |
|  | Rodent (Acomys spp, Arvicanthis, Gerbillus spp.) | [40] |
| * No reported vector for donovani in CL endemic areas | | |

**Supplementary Table 3:** Number of confirmed cutaneous leishmaniasis cases treated across the treatment centers in Ethiopia from 2017 to 2024, WHO

| **CL treatment center** | **Region** | **Zone** | **Woreda** | **Reporting Year** | | | | | | | |
| --- | --- | --- | --- | --- | --- | --- | --- | --- | --- | --- | --- |
|  |  |  |  | **2017** | **2018** | **2019** | **2020** | **2021** | **2022** | **2023** | **2024** |
| Alert | Addis Ababa | Addis Ababa | Kolfe Keraniyo | 1594 | 194 | 394 | 232 | 38 | 44 | 96 | 0 |
| Arba Minch Hospital | South Ethiopia Regional State | Gamo | A/Minch Town | 12 | 9 | 23 | 6 | 0 | 46 | 34 | 36 |
| Addis Alem Hospital | Ahmara | West Gojjam | Bahir Dar Town | 5 | 16 | 16 | 32 | 17 | 34 | 16 | 26 |
| Addis Zemen Hospital | Amhara | South Gondar | Addis Zemen Town | 47 | 98 | 42 | 77 | 81 | 55 | 44 | 71 |
| Ayder Hospital | Tigray | Mekelle | Mekelle town | 0 | 54 | 85 | 84 | 0 | 0 | 10 | 113 |
| Boru Meda Hospital | Amara | South Wello | Dessie Town | 41 | 135 | 495 | 240 | 157 | 213 | 0 | 816 |
| Chencha Hospital | South Ethiopia Regional State | Gamo | Chencha | 0 | 0 | 0 | 0 | 0 | 0 | 0 | 15 |
| Debre Birhan Hospital | Amhara | North Shewa (AM) | Debre Birhan Town | 0 | 0 | 0 | 0 | 0 | 0 | 0 | 6 |
| Denbi Dollo Hospital | Oromia | Kelem Wellega | Denbi Dollo town | - | 96 | 5 | 3 | 0 | 0 | 0 | 0 |
| Finote Selam Hospital | Amhara | West Gojam | Finote Selam Town | 11 | 39 | 24 | 11 | 5 | 37 | 21 | 12 |
| Gondar University Hospital | Amhara | Central Gondar | Gondar town | 88 | 110 | 83 | 67 | 107 | 227 | 93 | 170 |
| Nefas Mewch Hospital | Amhara | South Gondar | Nefas Mewch Town | - | - | 87 | 143 | 168 | 140 | 27 | 15 |
| Nekemt Hospital | Oromia | East Wolega | Nekemt town | - | - | 3 | 0 | 0 | 0 | 0 | 0 |
| Saint Mary Hospital | Tigray | Central | Axum Town | 0 | 1 | 0 | 5 | 0 | 0 | 1 | 0 |
| Selam Ber Hospital | South Ethiopia Regional State | Gamo | Kucha | 0 | 0 | 0 | 0 | 0 | 0 | 0 | 4 |
| Shedeho Meket Hospital | Amhara | North Wello | Meket | 0 | 0 | 0 | 0 | 0 | 0 | 0 | 8 |
| Tefera Hailu Hospital | Amhara | Wag Hamra | Sekota Town | 9 | 27 | 16 | 19 | 2 | 20 | 4 | 29 |
