# Supplementary figures and images for: The ecoepidemiology of cutaneous leishmaniasis in Ethiopia: a systematic review and meta-analysis
Source: Parasit Vectors. 2026 May 9;19:241. doi: 10.1186/s13071-026-07376-3 (PMC13238102; doi:10.1186/s13071-026-07376-3)

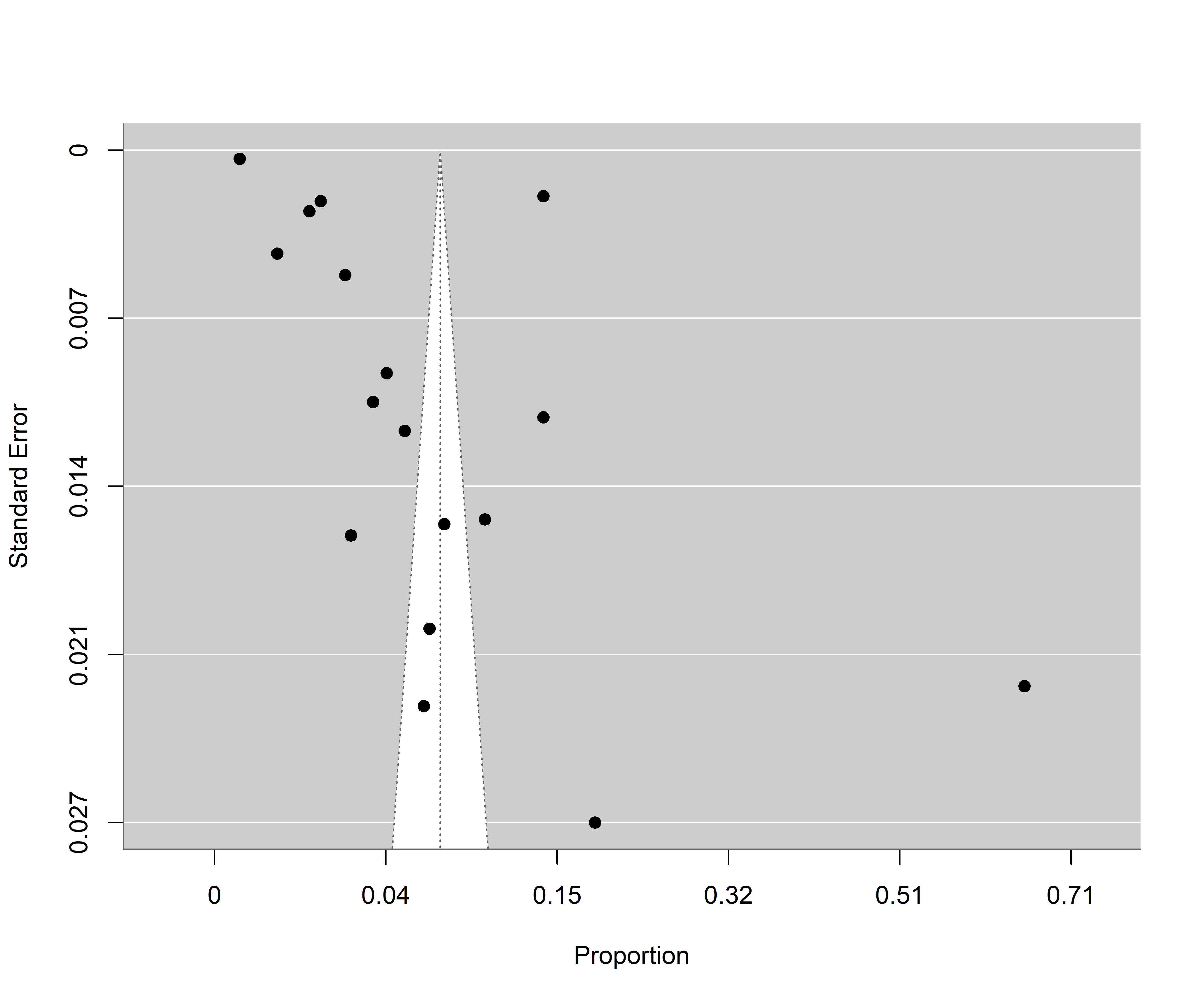

Supplement: Supplementary file 2 — Additional file 2: Figure S1. Funnel plot for assessing publication bias in the meta-analysis of proportions for the prevalence of cutaneous leishmaniasis in Ethiopia, 2025. [file 13071_2026_7376_MOESM2_ESM.tiff]

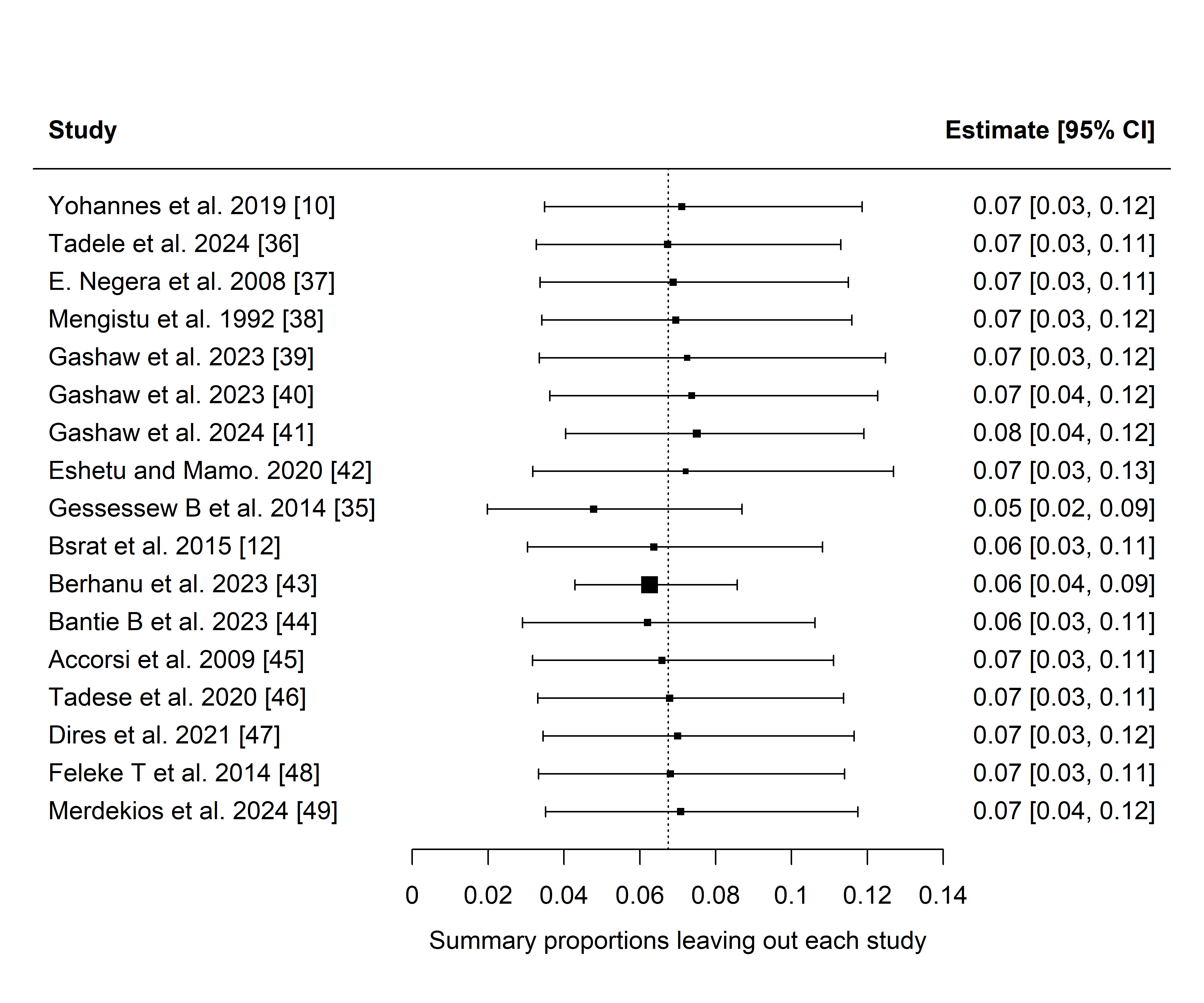

Supplement: Supplementary file 3 — Additional file 3: Figure S2. Leave-one-out forest plot to visually inspect the influence of outlier studies on the pooled proportion, for the prevalence of cutaneous leishmaniasis in Ethiopia, 2025. [file 13071_2026_7376_MOESM3_ESM.tiff]
